# Mycobacterium kansasii in cancer patients: a 13-case series

**DOI:** 10.1590/S1678-9946202668029

**Published:** 2026-04-10

**Authors:** Patrícia Rodrigues Bonazzi, Giovanni Mollo Baia, William Kazunori Sekiguchi, Adriana Satie Gonçalves Kono Magri, Raquel Keiko de Luca Ito, Karim Yaqub Ibrahim, Odeli Nicole Encinas Sejas, Edson Abdala

**Affiliations:** 1 Universidade de São Paulo, Faculdade de Medicina, Instituto do Câncer do Estado de São Paulo, São Paulo, São Paulo, Brazil; 2 Universidade de Mogi das Cruzes, Faculdade de Medicina, Mogi das Cruzes, São Paulo, Brazil; 3 Universidade de São Paulo, Faculdade de Medicina, Hospital das Clínicas, São Paulo, São Paulo, Brazil; 4 Universidade de São Paulo, Faculdade de Medicina, Departamento de Infectologia e Medicina Tropical, São Paulo, São Paulo, Brazil; 5 Universidade de São Paulo, Faculdade de Medicina, Instituto de Medicina Tropical de São Paulo, Laboratório de Hepatologia por Vírus (LIM-47), São Paulo, São Paulo, Brazil

**Keywords:** Mycobacterium kansasii, Cancer, Oncology, Nontuberculous mycobacterium

## Abstract

*Mycobacterium kansasii* comprises one of the most common and most pathogenic nontuberculous mycobacteria, particularly in immunocompromised individuals. This retrospective observational study aimed to describe the epidemiology, clinical characteristics, and outcomes of *M. kansasii* infections in 13 cancer patients from Instituto do Cancer do Estado de Sao Paulo, Brazil, from January 2011 to September 2023. Of the 19 initial positive cultures, 13 fully met diagnostic criteria for *M. kansasii* infections. Solid tumors occurred the most (69.2%), and only 38.4% of patients were undergoing chemotherapy at diagnosis. Smoking was the most frequent previous characteristic in these cases (76.9%). Fever (53.4%), cough (46.1%) and dyspnea (30.7%) were the most common symptoms. Multiple nodules (46.1%) and cavities (46.1%) were the most common radiological findings. Overall, seven patients (53.8%) received at least 12 months of therapy. One-year overall mortality totaled 38.4-22.2% in patients with solid tumors and 75% in patients with hematologic malignancies. In conclusion, *M. kansasii* infections were predominantly pulmonary in this series of cancer patients, which showed a high frequency of other respiratory comorbidities, especially smoking. Mortality occurred in several patients, particularly among those with hematological malignancies.

## INTRODUCTION

*Mycobacterium kansasii* is one of the most common nontuberculous mycobacteria (NTM) human pathogens, along with the *M. abscessus* and *M. avium* complexes^1^. These slow-growing mycobacteria take more than seven days to form colonies. In humans, the most common clinical presentations refer to lung infections and lymphadenitis. NTM infections occur more frequently in immunocompromised hosts.

*M. kansasii*, one of the most virulent NTM species,^1^ is the second most described in South America and sixth worldwide, although it is difficult to precisely determine its epidemiology since they cause non-reportable infection^2^. That is one of the reasons why it is fundamental to study this pathogen and how it presents on different groups.

Recent studies have investigated the clinical features of *M. kansasii* along with other NTM infections in people living with HIV/AIDS. In this scenario, it presents predominantly as a pulmonary infection in late-stage AIDS^3^.

Data regarding NTM infections in cancer patients remain limited, necessitating more reports to understand their epidemiological and clinical aspects. Most reports indicate *M. avium* complexes, that mainly show pulmonary involvement, with infection progression associated with advanced cancer and chemotherapy^4-6^. Jacobson KL et al. have reported *M. kansasii* infections in 25 cancer patients, observing an apparent association with leukemia and pulmonary radiologic findings with a predominance of multilobar infiltrates without cavitation^7^.

This study aims to describe the epidemiology, clinical features, and outcomes of *M. kansasii* infections in a series of 13 cancer patients.

## MATERIALS AND METHODS

This observational, retrospective, and descriptive study with a case series of *M. kansasii* in cancer patients was carried out at Instituto do Cancer do Estado de Sao Paulo, Sao Paulo State, Brazil, a tertiary cancer center. We found the patients at the center, with positive cultures for NTM from January 2011 to September 2023.

An initial search was made with the microbiology and infectious disease department database, in which all mycobacteria cultures of patients from Instituto do Cancer do Estado de Sao Paulo are recorded and monthly updated. Then, the cases with positive cultures for *M. kansasii* were analyzed.

The cases of *M. kansasii* infections were then determined according to the diagnostic criteria from the Infectious Diseases Society of America^8^. For NTM pulmonary disease, the guideline includes clinical pulmonary or systemic symptoms, radiological signs (such as nodular or cavitary opacities on a chest radiograph or a high-resolution computed tomography scan that shows bronchiectasis with multiple small nodules), and microbiologic criteria: positive cultures from at least two separate expectorated sputum samples, at least one bronchial wash or lavage, or from transbronchial or other lung biopsy with mycobacterial histologic features (granulomatous inflammation or acid-fast bacilli), positive cultures for NTM or biopsy showing mycobacterial histologic features (granulomatous inflammation or acid-fast bacilli) and one or more sputum or bronchial washings that are culture positive for NTM. All cases should meet all these three clinical, radiological, and microbiological criteria. Any respiratory symptom and/or fever were considered as clinical signs and symptoms. Overall, two infectious disease physicians from the group of authors reviewed the medical records, radiological reports, and microbiological data of the 19 cases initially identified with positive cultures for *M. kansasii*. In cases of no consensus, a third infectious disease physician, also an author of the study, was consulted. The following variables were obtained from patients’ electronic charts and evaluated: age, sex, oncological diseases, underlying diseases, chemotherapy (last six months), neutropenia (<500 cells/mm³ or <1000 cells/mm^3^ expecting further decrease within seven days), infection site, treatment, and outcomes. Death in one year after the last day of treatment was chosen as the evaluated outcome. Causes of deaths were ignored in this study.

### Ethics

This study was approved by the Comissao de Ensino e Pesquisa (CCEP) of the Instituto do Cancer do Estado de Sao Paulo, process Nº 5397/25, in August 19, 2025.

## RESULTS

**Figure 1:**
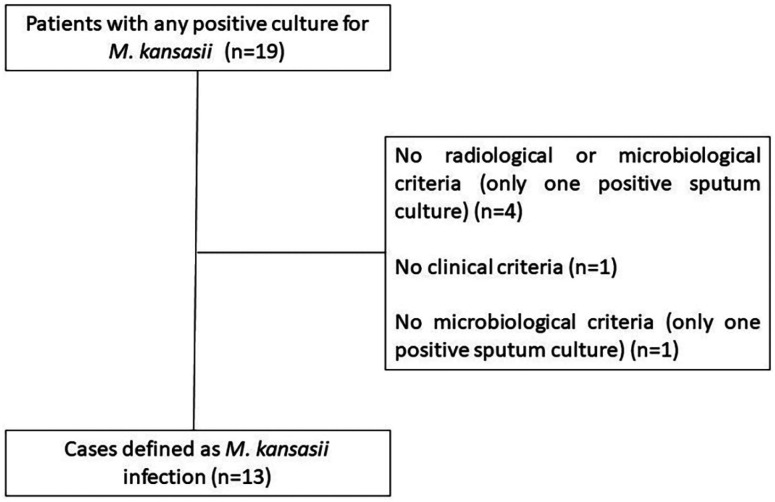
Flowchart for selecting and including patients with cancer and *M. kansasii* infection.

We firstly identified 19 consecutive positive cultures for *M. kansasii* during the studied period. After evaluating each individual patient record, 13 cases fully met the Infectious Diseases Society of America criteria for *M. kansasii* infection (Figure 1). Tables 1 and 2 show patients’ demographic, epidemiological, and clinical characteristics.

**Table 1:** Demographic, epidemiological, and clinical characteristics of 13 cancer patients with *M. kansasii* infection.

Patient	Sex	Age (years)	Oncological diagnosis	Chemo (last 6m)	Chemo scheme	Neutropenia	Comorbidities	Smoking
1	M	44	CML	Yes	Nilotinibe	No	Congenital hearing deficit	No
2	F	65	Cervix carcinoma	No	-	No	SAH, rheumatoid arthritis	Yes
3	M	61	Larynx SCC	No	-	No	AUD, pulmonary TB*	Yes
4	F	60	CML	Yes	Imatinibe	Yes	-	Yes
5	M	81	Rectum adenocarcinoma	No	-	No	SAH, pacemaker	Yes
6	M	66	AML	Yes	I3A7	Yes	Psoriasis	Yes
7	M	41	Non-Hodgkin lymphoma	No	-	NA	-	No
8	M	69	Lung SCC	Yes	Docetaxel	No	AUD	Yes
9	F	80	Larynx SCC	No	-	No	SAH, AUD, partial gastrectomy	Yes
10	F	45	Breast adenocarcinoma	Yes	AC-T	No	Fibromyalgia	Yes
11	M	66	Prostate adenocarcinoma	No	-	No	TB**	Yes
12	M	62	Cholangiocarcinoma	No	-	NA	SAH, AUD, Chronic hepatitis B	Yes
13	F	69	Esophagus SCC	No	-	NA	SAH, hypothyroidism	No

M = male; F = female; CML = chronic myeloid leukemia; SCC = squamous cell carcinoma; AML = acute myeloid leukemia; I3A7 = Idarubicin + cytarabine; AC-T = Doxorubicin/cyclophosphamide + Paclitaxel; SAH = systemic arterial hypertension; AUD = alcohol use disorder; TB = tuberculosis;

* treated in 2019;

** treated in 1995.

**Table 2:** Clinical and radiological characteristics and outcome of 13 *M. kansasii* infection in cancer patients

Patient	Site of infection	Positive culture specimen	Clinical symptoms	Radiological signs	Treatment	Treatment - Drugs	Treatment - Duration	Outcome
1	Systemic	1- Blood / 2- Cervical lymph node biopsy	Fever, cough, nausea	Multiple bilateral pulmonary small nodules	Yes	RHZE	12 months	Alive
2	Disseminated - Lymph nodes/ pulmonary	1 - Lymph nodes / 2 - Sputum	Fever, cough, inguinal pain	Multiple bilateral pulmonary consolidation areas	Yes	RHEC	12 months	Alive
3	Pulmonary	1- BAL / 2- Lymph node	Cough	Multiple bilateral pulmonary nodules, 1 cavitary. Mediastinal lymphadenopathy	Yes	RHZE	15 months	Alive
4	Pulmonary	1- BAL/ 2- Sputum	Dyspnea	Superior pulmonary right lobe consolidation with cavitary area. Pleural effusion. Mediastinal lymphadenopathy.	Yes	RHZE-C	3 days	Death
5	Pulmonary	1- Sputum / 2 - Sputum	Fever, fatigue	Multiple bilateral pulmonary nodules. Mediastinal lymphadenopathy	Yes	RHEC	12 months	Alive
6	Pulmonary	1- Sputum / 2- Sputum	Fever, weight loss, dyspnea	Inferior pulmonary right lobe consolidation. Pleural effusion	No	-	-	Death
7	Pulmonary	1- BAL/ 2- Sputum	Fever, cough	Multiple bilateral pulmonary nodules	No	-	-	Death
8	Pulmonary	1- Sputum/ 2- Sputum	Fever, dyspnea,	Multiple bilateral pulmonary nodules. Mediastinal lymphadenopathy	No	-	-	Death
9	Pulmonary	1- Sputum/ 2- Sputum	Fever, dyspnea	Superior pulmonary left lobe consolidation with cavitary area	No	Clarithromycin + Imipenem	8 days	Death
10	Pulmonary	1- BAL/ 2- Sputum	Fever, cough, weight loss	Pulmonary cavitary formation in superior right lobe	Yes	RHE	18 months	Alive
11	Lymph nodes	1- Lymph nodes	Cough, weight loss	Multiple bilateral pulmonary nodules. Mediastinal lymphadenopathy	Yes	RHE	18 months	Alive
12	Pulmonary	1- BAL	Fever, weight loss	Pulmonary cavitary lesion in superior left lobe	No	-	-	Alive
13	Pulmonary	1- BAL/ 2- BAL	Fever	Pulmonary cavitary formation in superior right lobe	Yes	RHZE (2 months) => RHE (16 months) + Streptomycin (12 months)	18 months	Alive

BAL = bronchoalveolar lavage; RHZE = rifapentine + Isoniazid + pyrazinamide + ethambutol; RHEC = rifapentine + Isoniazid + ethambutol + capreomycin; RHZE-C = RHZE + capreomycin; RHE: rifapentine + Isoniazid + ethambutol.

Patients were mostly men (61.5%), with a mean age of 62.2 years. Patients varying oncological diagnoses, with a predominance of solid tumors (69.2%). Most participants had received no chemotherapy in the six months prior to diagnosis (61.5%). The most frequent pre-existing characteristics were smoking (76.9%) and systemic arterial hypertension (SAH) (38.4%). Neutropenia occurred in only two (15.4%) patients.

Most patients had pulmonary infections (84.6%). Of all the described symptoms, fever (53.4%), cough (46.1%) and dyspnea (30.7%) occurred the most often. As for radiological findings, multiple nodules (46.1%) and cavities (46.1%) were the most common.

In total, seven patients (53.8%) received at least 12 months of adequate treatment. Mortality totaled 38.4% (5 cases), of whom none had received a minimum period of adequate treatment. Also, three of those cases were diagnosed postmortem.

## DISCUSSION

Little data are available in the current literature about *M. kansasii* infections in oncological patients. Our study brings some new additional information in this scenario that may add to such scarce data.

Most of our patients had solid tumors (69.2%). We observed a majority of pulmonary infections and older patients, concurring with recent studies^1^. Most patients smoked and had respiratory comorbidities (76.9%), and two patients had a history of pulmonary tuberculosis (TB), (ignoring those with lung or airway cancer). In fact, in this series, we found a higher frequency of association between the infections and previous respiratory and pulmonary conditions than with oncological diseases and their respective treatments. Only five of the 13 patients (38.4%) were undergoing chemotherapy at the time of the diagnosis, and only two had neutropenia. However, we are unable to determine a risk relationship since we performed no risk factor analysis.

Of all the described symptoms, fever 53.4%), cough (46.1%) and dyspnea (30.7%) were the most common. As for radiological findings, multiple nodules (46.1%) and cavities (46.1%) occurred the most often. This is mostly consistent with the literature, except for the lack of hemoptysis, but overall, it is very similar to TB^8^.

NTM infections usually show nonspecific radiological findings and symptoms, challenging diagnostic suspicions. Unlike TB, the diagnosis of NTM infection requires clinical, radiological, and microbiological criteria^9,10^. The need for diagnostic precision can sometimes delay diagnosis and the adoption of therapeutic measures.

Just like diagnosis, the treatment for *M. kansasii* infections can offer a challenge. The decision to start treatment heavily relies on accurate diagnoses, and the recommended regimen and treatment schemes vary a lot, again different from TB. In general, the recommended treatment is to last at least 12 months. We observed that in some cases in our series the initial therapy included treatment for TB. Probably, at that time, the diagnosis of *M. kansasii* infection was yet to be definitively established.

Only seven patients (53.8%) received at least 12 months of treatment. Among the others, three received no treatment and two received very short treatments: three and eight days respectively. These last five patients were precisely the cases of death. Although we included these cases in the mortality assessment, their deaths occurred so early in the course of their disease that no association can be inferred between treatment and clinical outcome. We must also consider a survival bias since patients who survived had time to receive treatment and those who died early were unable to.

In this descriptive case series, deaths predominantly occurred in patients with hematologic malignancies, although we are unable to make comparative inferences. Of the five patients who died, three were undergoing chemotherapy and two had neutropenia. These factors could be associated with a worse and quicker evolution, prohibiting the needed time for diagnosis and treatment. Therefore, although chemotherapy and neutropenia may have constituted no risk factor for the *M. kansasii* infections, they might be related to worse prognoses. Similarly, Nqwata *et al.* ^11^ observed a higher mortality in patients with HIV and NTM infections with a higher degree of immunosuppression (CD4 < 50 cells/m^3^) than in those with a CD4 > 50 cells/m^3^.

We found that one patient that survived without receiving treatment. They received no specific treatment for *M. kansasii*. However, they had multiple other infectious complications (not only in their respiratory system) and received various antibiotic schemes in intensive care. We suspect that this association may have made an effect, substituting the usual treatment.

### Limitations

Our study has some limitations. This single center and retrospective study had a small number of cases, heterogeneous oncologic conditions and possible survival bias since patients who survived longer had time to receive therapy, whereas those who died early were unable to. On the other hand, given the scarce data available on *M. kansasii* infections in cancer patients, our cases and analysis can contribute with relevant and useful information on how to manage these patients.

## CONCLUSION

In conclusion, this series of cancer patients predominantly had pulmonary *M. kansasii* infections. It also showed a high frequency of other respiratory comorbidities, especially smoking, in such cases. Mortality occurred in several patients, particularly among those with hematological malignancies.

## Data Availability

The anonymized dataset generated during this study is available from the corresponding author upon reasonable request.
